# Aureusidin derivative CNQX inhibits chronic colitis inflammation and mucosal barrier damage by targeting myeloid differentiation 2 protein

**DOI:** 10.1111/jcmm.16755

**Published:** 2021-06-29

**Authors:** Yi Yang, Yongjia Sheng, Jin Wang, Xiaohong Zhou, Qiaobing Guan, Heping Shen, Wenyan Li, Shuiliang Ruan

**Affiliations:** ^1^ Department of Pharmacy The Second Affiliated Hospital of Jiaxing University Jiaxing China; ^2^ Department of Center Laboratory The Second Affiliated Hospital of Jiaxing University Jiaxing China

**Keywords:** chronic colitis, inflammatory response, mucosal barrier, myeloid differentiation 2

## Abstract

Our previous study has found that aureusidin can inhibit inflammation by targeting myeloid differentiation 2 (MD2) protein. Structural optimization of aureusidin gave rise to a derivative named CNQX. LPS was used to induce inflammation in intestinal macrophages; flow cytometry, PI staining and Hoechst 33342 staining were used to detect the apoptotic level of macrophages; enzyme‐linked immunosorbent assay (ELISA) was utilized to detect the expression level of inflammatory factors (including IL‐1β, IL‐18 and TNF‐α); immunofluorescence staining was used to investigate the expression of MD2; Western blot was employed to measure the protein level of TLR4, MD2, MyD88 and p‐P65. As a result, CNQX with IC50 of 2.5 μM can significantly inhibit the inflammatory damage of macrophages, decrease apoptotic level, reduce the expression level of inflammatory factors and simultaneously decrease the expression level of TLR4, MD2, MyD88 as well as p‐P65. Caco‐2 cell line was used to simulate the intestinal mucosal barrier in vitro, LPS was employed to induce cell injury in Caco‐2 (to up‐regulate barrier permeability), and CNQX with IC50 of 2.5 μl was used for intervention. Flow cytometry was used to detect the apoptotic level of Caco‐2 cells, trans‐epithelial electric resistance (TEER) was measured, FITC‐D was used to detect the permeability of the intestinal mucosa, and Western blot was used to detect the expression levels of tight junction proteins (including occludin, claudin‐1, MyD88, TLR4 and MD2). As a result, CNQX decreased the apoptotic level of Caco‐2 cells, increased TEER value, decreased the expression levels of MyD88, TLR4 and MD2, and increased the protein levels of tight junction proteins (including occludin and claudin‐1). C57BL/6 wild‐type mice were treated with drinking water containing Dextran sulphate sodium (DSS) to establish murine chronic colitis model. After CQNX intervention, we detected the bodyweight, DAI score and H&E tissue staining to evaluate the life status and pathological changes. Immunohistochemistry (IHC) staining was used to detect the expression of MD2 protein, tight junction protein (including occludin and claudin‐1). Transmission electron microscopy and FITC‐D were used to detect intestinal mucosal permeability. Western blot was used to detect the expression levels of tight junction proteins (including occludin, claudin‐1, MyD88, TLR4 and MD2) in the intestinal mucosa tissue. Consequently, CNQX can inhibit the intestinal inflammatory response in mice with colitis, inhibit the mucosal barrier injury, increase the expression of tight junction proteins (including occludin and claudin‐1) and decrease the expression levels of MyD88, TLR4 and MD2. Mechanistically, pull‐down and immunoprecipitation assays showed that CNQX can inhibit the activation of TLR4/MD2‐NF‐κB by binding to MD2 protein.

Collectively, in this study, we found that CNQX can suppress the activation of TLR4 signals by targeting MD2 protein, thereby inhibiting inflammation and mucosal barrier damage of chronic colitis.

## 1 BACKGROUND

Inflammatory bowel disease (IBD) is an idiopathic, chronic and recurrent intestinal inflammatory disease involving the ileum, rectum and colon,^1^ including Crohn's disease (CD) and ulcerative colitis (UC), which is clinically manifested as diarrhoea, abdominal pain, bloody stools, weight loss, even with severe complications.^2^, ^3^ IBD was relatively more common in Western developed countries in the past. In recent years, the incidence of IBD has shown a linear upward trend in Asia, including China. Epidemiological studies have shown that the incidence of IBD in China is as high as 3.44%, ranking the first in Asia.^4^ The aetiology and pathogenesis of IBD have not been fully understood yet. At present, it is generally believed that IBD is caused by various factors and is affected by the interaction of environmental, genetic, infection and immune factors. Among them, immune factors are considered as one of the important factors causing IBD.^5^ At present, it is believed that the disorder of the mucosal barrier function is the main cause of IBD. The antigen stimulates the intestinal mucosa injury to increase the permeability of the mucosa to stimulate the production of various inflammatory factors.^6^, ^7^ Myeloid differentiation 2 (MD2) is a complex that binds to Toll‐like receptor 4 (TLR4) and mediates LPS‐induced inflammatory response, playing an important role in sepsis and malignancies.^8^ Studies have revealed that TLR4/MD2 receptors can cause the release of downstream inflammatory factors after receiving upstream signals, thereby initiating the inflammatory response.^9^, ^10^ Our team have previously demonstrated that MD2 protein is highly expressed in IBD, and MD2 is involved in the pathogenesis and progression of IBD, playing an important role in mucosal barrier damage.^11^, ^12^ Intervention with MD2 protein can block TLR4 signal transduction and inhibit mucosal barrier damage in IBD.

Curcumin and its analogs can be used as MD2 targeted binding small molecule compounds to inhibit the inflammatory response of sepsis, and the analog structure is a diketone compound.^13^ Aureusidin is an active substance extracted from antirrhinum majus, which belongs to orange ketone compounds in structure. The preliminary study of our group has found that aureusidin is a small molecule targeting MD2 through screening and research. Through liver injury models, pull‐down and immunoprecipitation assays have shown that aureusidin can inhibit the activation of TLR4/MD2‐NF‐κB by binding to the MD2 protein.^14^ Further structural modification identified that an open‐loop derivative CNQX exerted a significant anti‐inflammatory effect. Therefore, in this study, we focussed on the relevant and mechanism of CNQX on IBD.

## 2 MATERIAL AND METHODS

### 2.1 Cell culture and intervention of CNQX

Intestinal macrophages (Wuhan Procell Biotechnology Co., Ltd.) were recovered and cultured in complete medium at 37℃ in a humid incubator containing 5% CO_2_. After detecting cell viability by trypan blue staining, cells in the logarithmic phase were divided into DMSO group, LPS group and CNOX treatment groups. Macrophages in the LPS group were stimulated with LPS of 0.5 mg/L to induce inflammation. CNQX with IC50 of 2.5 μM for 6 h and 1 mg/L LPS was used to induce inflammation.

### 2.2 Effect of CNQX on cell viability and apoptosis

#### 2.2.1 Detection macrophage viability by CCK‐8 assay

Intestinal macrophages were seeded into 96‐well plates. Three replicates were set in each group, followed by detection every 6 h within 24 h after LPS intervention. Cells were added with 10 μl of CCK‐8 reagent (Beyotime Biotechnology Co., Ltd.) and incubated for 2 h, followed by detection of the absorbance at a wavelength of 450 nm.

#### 2.2.2 Detection of cell apoptosis by flow cytometry

Intestinal macrophages were inoculated into 6‐well plates and stimulated with LPS for 12 h. Both suspended and adherent cells were collected, washed with PBS for two times, resuspended in pre‐cooled PBS. After centrifugation, cells were suspended in binding buffer and subjected to the cell apoptosis detection kit (BD, Massachusetts, USA) was used for detection. 5 μl of Annexin V‐FITC and 5 μl of PI staining were added to the cell suspension and subjected to flow cytometry after incubation in dark for 15 min.

#### 2.2.3 Detection of the apoptotic level by PI staining and Hoechst 33342 staining

Intestinal macrophages were inoculated into the 6‐well plate, stimulated with LPS for 12 h and washed with PBS for two times. Cells were incubated with Hoechst 33258 staining solution (Beyotime Biotechnology Co., Ltd.,) for 15 min and washed with PBS for two times. The positive cells showed blue fluorescence under microscope. For PI staining, cells were stained with PI staining reagent (Beyotime Biotechnology Co., Ltd.) at 1 μg/ml for 30 min, and washed with PBS for two times. Positive cells showed red fluorescence under microscope.

### 2.3 The effect of CNQX on the expression of inflammatory factors and related proteins

#### 2.3.1 Detection of the expression levels of inflammatory factors (including IL‐1β, IL‐18 and TNF‐α) by ELISA

Intestinal macrophages were inoculated into the 12‐well plate and stimulated with LPS for 12 h and 24 h. The cell culture medium was collected after LPS treatment and subjected to the ELISA kit (Nanjing Jiancheng Institute of Biology) to measure the levels of inflammatory factors according to the manufacturer's instruction (results shown as pg/ml).

#### 2.3.2 Detection of the expression level of MD2 by immunofluorescence (IF) staining

The cover glass was placed in the 6‐well plate. Intestinal macrophages were inoculated and stimulated with LPS for 12 h, fixed with 4% paraformaldehyde (PFA) for 10 min, washed with PBS for three times and permeabilized with 0.2% Triton X‐100 for 10 min, blocked with 2% BSA for 30 min, incubated with MD2 monoclonal antibody (Abcam dilution 1:300) for 1 at room temperature, incubated with IgG antibody (Abcam) for labelling, added with 0.5 μg/ml DAPI staining reagent (Solarbio) for nuclear staining, washed with PBS for two times, mounted and observed under fluorescence microscope.

#### 2.3.3 Detection of the expression level of key proteins by Western blot

Cells were inoculated into the 6‐well plate and stimulated with LPS for 12 h. The collected cells were washed with PBS for two times, added with 1.0 ml of RIPA lysis buffer (Beyotime Biotechnology Co., Ltd.) and lysed on ice for 30 min. After protein quantification, protein sample was subjected to electrophoresis and transferred to the PVDF membrane. The membranes were blocked with 5% skimmed milk powder for 2 h, incubated with primary monoclonal antibodies (including TLR4, MD2, MyD88 and p‐P65) (Abcam, dilution 1:500) diluted in TBST. The blot was subsequently reacted with HRP‐labelled goat anti‐rabbit secondary antibody (Abcam, dilution 1:2000). After incubation, the membranes were visualized with ECL, followed by optical density analysis by Image‐Pro Plus 6.0 software. GAPDH was used as the internal control, and the results were shown as the comparison of the optical density value between the target protein and the internal control protein.

### 2.4 Intervention of CNQX on Caco‐2 cell mucosal barrier damage in vitro

Caco‐2 cells (Wuhan Procell Biotechnology Co., Ltd.) were recovered and cultured in complete medium. After detecting cell viability by trypan blue staining, cells were divided into DMSO group, LPS group and CNQX group. Macrophages in the LPS group were stimulated with LPS of 0.5 mg/L to induce inflammation. Pre‐treatment of CNQX with IC50 of 2.5 μM for 6 h and 1 mg/L LPS were used to induce inflammation. The procedures of flow cytometry to detect the apoptotic level and Western blot to detect the level of protein were the same as the intestinal macrophage.

### 2.5 Detection of Caco‐2 Mucosal Barrier Injury

#### 2.5.1 Transepithelial electrical resistance (TEER) measurement

Caco‐2 cells were seeded in Transwell chamber at a density of 4 × 10^5^ cells/ 1 mL (200 μL per well). Moreover, 600 μL of 1640 culture medium was added to the lower chamber. After forming compact cell layer, cells were divided and interfered for 12 h, followed by TEER measurement. TEER was determined by Millicell resistance meter. The whole process was performed at 37℃. Three points in different directions were randomly selected for each Transwell chamber, and the measurement was repeated three times. The resistance value was expressed in ohm/cm^2^ (Ω/cm^2^). Standard TEER value = (measured value‐measured value of blank control)/0.33 cm^2^.

#### 2.5.2 Detection of FITC‐D permeability

FITC‐D was used as a marker to detect the transport effect of Caco‐2 cells. After stimulation of LPS for 12 h, cells were carefully rinsed with Hank's Balanced Salt Solution (HBSS) for three times. FITC‐D at a final concentration of 1 mg/mL was added to the top Transwell chamber, and 0.6 mL HBSS was added to the lower chamber. The liquid in the lower chamber after incubation at 37℃ for 1 h. Fluorescence intensity was measured by fluorescence spectrophotometer (excitation wavelength 490 nm, emission wavelength 520 nm), and FITC‐D concentration was calculated according to the FITC‐D standard curve. FITC‐D permeability (%/h/cm^2^) = (FITC‐D fluorescence value of the lower chamber/FITC‐D fluorescence value initially added to the top chamber) /1 h/0.33 cm^2^ × 100%

### 2.6 Intervention of CNQX on mice with chronic colitis

Wild‐type C57BL/6 mice (weight of 20‐22 g) were adapted for one week and randomly divided into control group, model group and CNQX group (10 mice in each group). Mice in the model group and CNQX group were fed 2.5% DSS to establish chronic colitis mice. Mice in the CNQX group were administered with 10 mg/kg CNQX by gavage once a day. Mice were treated with drinking water containing 2.5% DSS at 1‐5, 8‐12, 15‐19 and 22‐26 days, and treated with distilled water for the rest of the time (a total of four cycles). The DAI score and weight loss rate of mice were measured on the first 1, 3 and 5 days of each cycle. On the 29th day, mice were sacrificed by deep anaesthesia. The skin and muscle layer of the abdominal cavity were quickly resected, followed by separation from the terminal ileum to the rectum. The tissue was washed with PBS (Sigma) for three times, and the colon tissue was fixed with 4% PFA.

### 2.7 Evaluation of bodyweight, DAI score and pathological conditions of mice with chronic colitis

For bodyweight loss rate of mice, the bodyweight was measured on days 1, 3 and 5 of each cycle, and the weight ratio was compared to the initial weight. DAI score was based on the pathological state of mice. According to the DAI score standard, the weight, foecal traits and occult blood of mice were evaluated and recorded on days 1, 3 and 5 of each cycle.

### 2.8 H&E pathological staining of intestinal tissue

The paraffin‐embedded colon tissue was cut into 4 μm serial sections, deparaffinized with xylene, dehydrated with 100%‐95%‐80% gradient ethanol, stained with haematoxylin for 3min, added with 1% hydrochloric acid alcohol for 2s, incubated with 1% ammonia water for 20s, treated with 0.5% eosin alcohol for 10s, dehydrated with gradient alcohol, transparented by xylene and mounted using neutral gum, followed by observation of the pathological changes of intestinal tissue under light microscope.

### 2.9 Detection of Intestinal Mucosal Barrier Damage in Mice

#### 2.9.1 Detection of the ultrastructure of intestinal villi by transmission electron microscopy (TEM)

The colon tissue placed in 2.5% pre‐cool glutaraldehyde fixative (for imported electron microscopy). The sample was cut into 1 mm^3^ in the fixative solution, transferred to a 1.5 ml tip EP tube and fixed in fill‐up fixative solution at 4℃ overnight, pre‐fixation: fix at 4℃ for 24 h, rinse with 0.1 M PBS for three times (10min each time), post‐fixation: 1% osmium acid, fix at 4℃ for 2 h, rinse with 0.1 M PBS for three times (10 min each time). Dehydration, soaking, embedding, staining and observation: 1% acetic acid axis staining for 20 min, lead citrate staining for 7 min, observation and image acquisition by TEM.

#### 2.9.2 FITC‐D detection of intestinal mucosal permeability

After the intervention was completed, mice were fasted and deprived of water for 4 h before sacrifice. Mice were treated with fluorescein isothiocyanate‐labelled dextrose dextran, FITC‐D, MW 4000) by gavage (60 mg FITC‐D /100 g), followed by serum collection. The fluorescence density of each sample was measured by fluorescence spectrophotometer, and the serum concentration of FITC‐D was measured.

### 2.10 Detection of proteins related to mucosal barrier damage in mice

#### 2.10.1 Detection of the expression of MD2 protein, tight junction protein (including occludin and claudin‐1) by IHC

Mouse intestinal tissue was fixed with 4% PFA, paraffin‐embedded and then sliced, baked at 60℃ for 2 h, treated with xylene and gradient alcohol. The slices were placed in 0.01 mol/L citrate buffer (PH = 6.0), heated at 98℃ in a microwave for 20 min for antigen retrieval, incubated with 3% hydrogen peroxide at room temperature for 10 min to eliminate endogenous peroxidase, blocked with 2% bovine serum albumin (BSA) at room temperature for 30 min, incubated with monoclonal antibodies against MD2, occludin and claudin‐1 (Abcam, dilution 1:300), washed with TBS for three times, added with proper secondary antibody at 37℃ for 15 min, incubated with peroxidase‐labelled streptomycin (Mexin Biotechnology Development Company) for 15 min, rinsed with PBS for three times (5 min each time), visualized with freshly prepared DAB solution (Dako), counterstained with haematoxylin and finally mounted. For negative control, primary antibody was replaced by TBS. The Olympus‐DP72 image acquisition system and the Olympus‐BX51 upright microscope of the CRi Nuance multispectral imaging system (Cambridge Research & Instrumentation) were used for image acquisition and quantitative analysis.

#### 2.10.2 Detection of the expression levels of tight junction protein (including occludin and claudin‐1), MyD88, TLR4 and MD2 in the intestinal mucosal tissue by Western blot

The mouse colon tissue was ground in liquid nitrogen, which was further subjected to Western‐Blot analysis accordingly.

### 2.11 Determination of the target binding relationship between CNQX and MD2

#### 2.11.1 Virtual docking

MGLTools software was used to hydrogenate MD2 protein and to calculate the charge processing. Molecular docking (AutoDock Vina 1.1.2) was performed between the MD2 receptor protein and the CNQX ligand small molecule and the optimal conformation (−9.4 kcal/mol) was subjected to PyMOL to plot a three‐dimensional graph to show the hydrogen bond between receptor protein and small ligand molecule. In addition, Ligplus software was used to plot a two‐dimensional graph to show the hydrophobic interaction between receptor protein and small ligand molecule.

#### 2.11.2 Bis‐ANS fluorescence spectrum assay

For the first time, 95 μl of PBS and 5 μl of bis‐ANS solution were added to the cuvette, followed by fluorescence detection using a microplate reader (excitation wavelength at 385 nm and emission wavelength at 430‐550 nm). For the second time to detect the absorption characteristics of MD2 protein, 90 μl of PBS, 5 μl of 0.1 mM bis‐ANS solution and 5 μl of 0.1 μM rhMD2 protein (Abcam) were added to the cuvette, followed by fluorescence detection after mixing. For the third time, 87.5 μl of PBS, 5 μl of 0.1 mM bis‐ANS solution and 5 μl of 0.1 μM rhMD2 protein and CNQX were added to the cuvette (the concentration of aureusidin was 1‐2.5‐5‐10 μM), followed by detection of fluorescence absorption after incubation at room temperature for 5 min.

#### 2.11.3 Co‐immunoprecipitation (Co‐IP) assay

Intestinal macrophages were inoculated into the 6‐well plate for CNQX intervention. Afterwards, cells were washed with PBS and lysed to collect protein. The TLR4 monoclonal antibody according to the ratio of 2 μL to 200 μg antibody and incubated at 4℃ overnight. The sample was further incubated with protein G agarose affinity matrix, at 4℃ in a shaker for 4 h. The supernatant was discarded and washed with pre‐cooled PBS for four times. Finally, the expression of TLR4 and MD2 was detected by Western blot.

#### 2.11.4 Co‐IP assay for biotin‐labelled CNQX

SUMO‐labelled MD2 protein was expressed in E. coli overnight. Cells were collected from suspension in Tris‐HCl and NaCl, sonicated, centrifuged and purified by Ni column. His‐SUMO‐MD2 protein was washed with 500mM imidazole for two times, His‐SUMO tag was cleaved by SUMO protease (ULP‐1) and purified with Ni column. The purified protein was separated by SDS‐PAGE. Subsequently, 15 μg of recombinant protein and biotin‐labelled CNQX (Biotin‐CNQX) was combined. After the Biotin‐CNQX incubation, the magnetic beads were washed with Tris buffer for three times and boiled with buffer and β‐mercaptoethanol. For MD2 pull‐down, the recombinant protein G magnetic beads and MD2 antibody (Abcam, Massachusetts, USA) were incubated. After washing with Tris buffer, MD2 protein was detected by Western blot, and HRP‐conjugated anti‐biotin antibody was utilized for biotin detection (CST).

#### 2.11.5 Surface Plasmon Resonance (SPR) assay

CNQX was prepared at 0.25‐0.74‐2.22‐6.67‐20 μM using PBSTD buffer, and the protein was fixed using the amino Eulink method. The 0.1 M Sulfo‐NHS and 0.4 M EDC were mixed to pass through the chip channel, 0.68 M rhMD2 acetate buffer solution also passed through the channel for activation, followed by ethanolamine hydrochloride treatment. After passing CNQX solution through the channel at 25℃, the signal was collected and processed with ProtenOn Manager 2.1.2 software.

### 2.12 Statistical analysis

The measurement data were expressed as mean ± standard deviation ($\bar{x}$ ± s). One‐way ANOVA was used for comparison between multiple groups, SNK test was used for comparison between groups, and SPSS 18.0 software was utilized for statistical analysis. A *P* < .05 was considered as statistical significance.

## 3 RESULTS

### 3.1 CNQX inhibited the inflammatory response of intestinal macrophages

**FIGURE 1:**
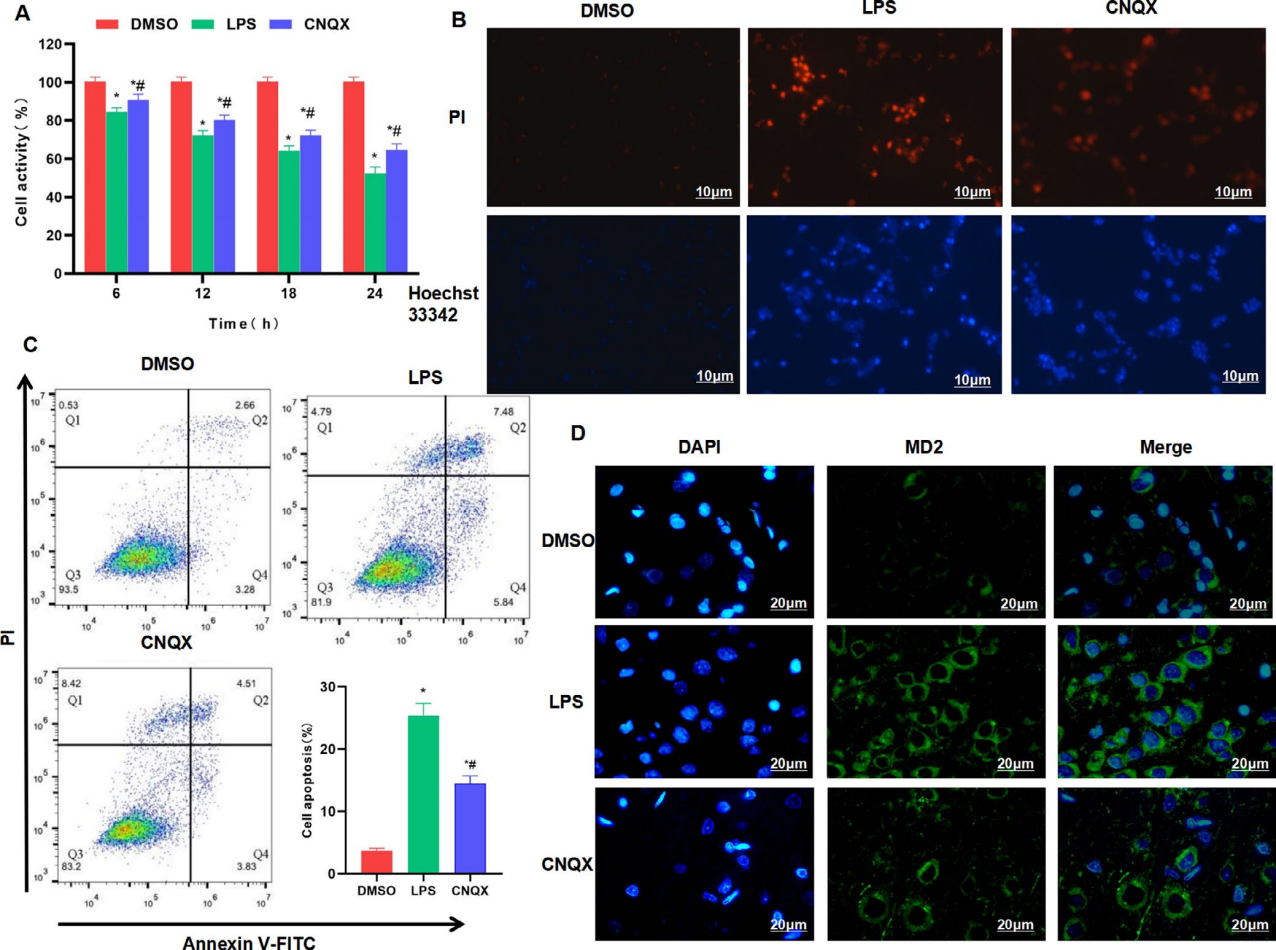
The effect of CNQX on the expression of MD2 protein, an inhibitor of intestinal macrophage inflammation (A) Cell viability by CCK‐8 assay ($\bar{x}$ ± s, n = 3): The cell viability in the LPS group was down‐regulated, which was significantly lower than that in the DMSO group, whereas the cell viability in the CNQX group was higher than that in the LPS group. Comparison with the DMSO group, ^*^ *P* < .05; comparison with the LPS group, ^#^ *P* < .05 at the same time‐point. (B) Detection of apoptotic level by PI and Hoechst 33342 staining (n = 3): The cell staining in the DMSO group was negative, whereas the number of positive cells in the LPS group was significantly up‐regulated and higher than that of the DMSO group. However, CNQX can inhibit cell apoptosis and decrease the number of positive cells. (C) Detection of apoptotic level by flow cytometry ($\bar{x}$ ± s, n = 3): LPS can induce apoptosis, and the rate of apoptosis in the LPS group was higher than that of the DMSO group, whereas CNQX can inhibit apoptosis and down‐regulate the apoptotic rate. Comparison with DMSO group, ^*^ *P* < .05; comparison with LPS group, ^#^ *P* < .05. (D) Immunofluorescence staining of MD2 protein (n = 3): The expression level of MD2 protein in the DMSO group was lower, and the fluorescence intensity was weak. The expression level of MD2 protein in the LPS group was higher, and the fluorescence intensity was stronger than that in the DMSO group. Moreover, CNQX can inhibit the MD2 protein and down‐regulate the fluorescence intensity

**FIGURE 2:**
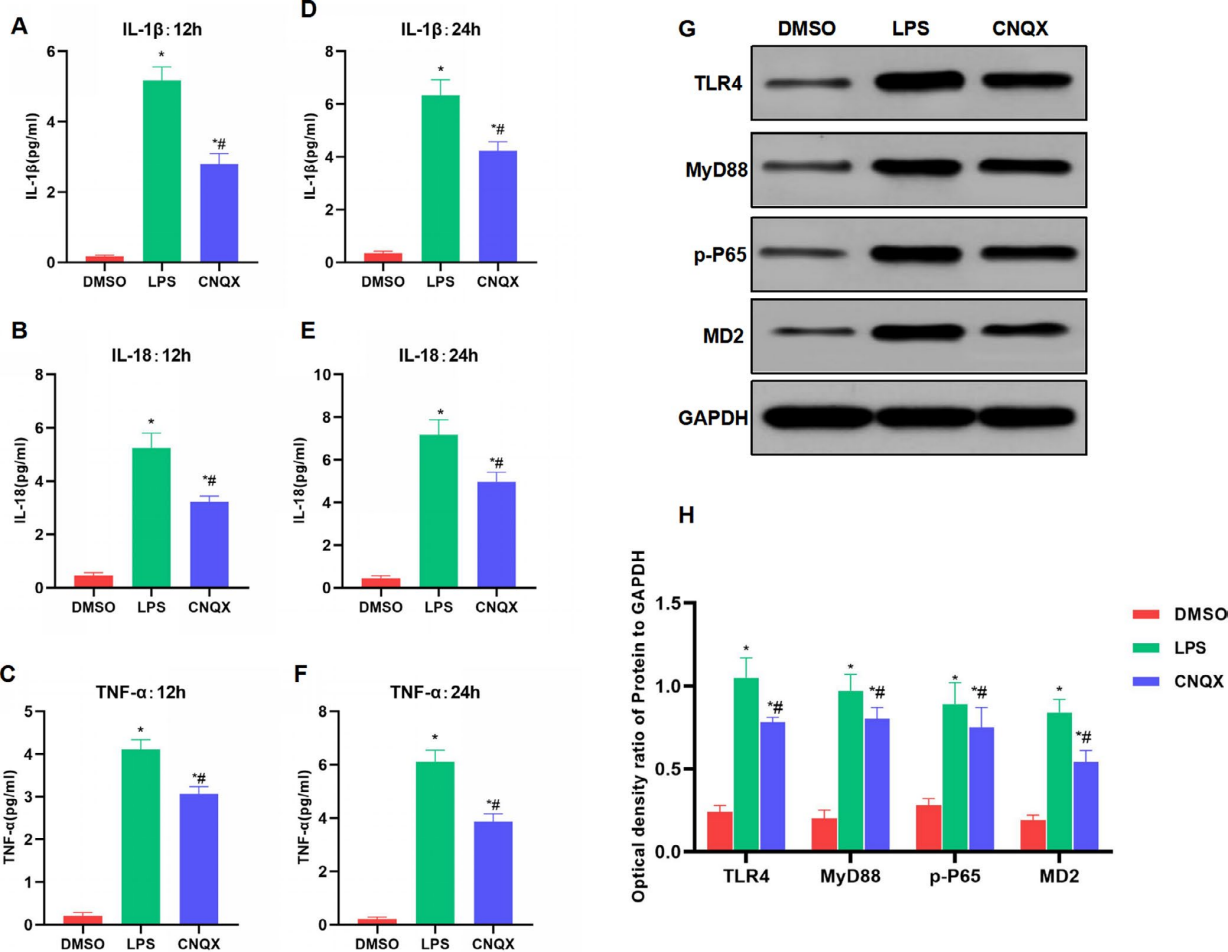
The effect of CNQX on the expression of inflammatory factors and key protein of TLR4 signal in intestinal macrophages (A‐C) The expression of inflammatory factors after 12h ($\bar{x}$ ± s, n = 3): The expression levels of inflammatory factors (including IL‐1β, IL‐18 and TNF‐α) were low in the DMSO group, and the expression levels were significantly up‐regulated in the LPS group. However, the expression levels of inflammatory factors in the CNQX group were down‐regulated, which were significantly lower than that in the LPS group. Comparison with DMSO group, ^*^ *P* < .05; comparison with LPS group, ^#^ *P* < .05. (D‐F) The expression of inflammatory factors after 24h ($\bar{x}$ ± s, n = 3): The results were similar to the results of 12h. The inhibitory effect on inflammation in CNQX group was significantly lower than that in LPS group. Comparison with DMSO group, ^*^ *P* < .05; comparison with LPS group, ^#^ *P* < .05. (G‐H) The expression of key protein of TLR4 signal ($\bar{x}$ ± s, n = 3). The TLR4 signal was activated, and the protein levels of TLR4, MyD88, MD2 and downstream p‐P65 were significantly up‐regulated in the LPS group, which were higher than those of the DMSO group. However, CNQX treatment can significantly inhibit the activation of TLR4 signal and down‐regulate protein levels. For relative protein expression, comparison with the DMSO group, ^*^ *P* < .05; comparison with the LPS group, ^#^ *P* < .05

The CNQX treatment with IC50 of 2.5 μM can significantly inhibit the inflammatory response induced by LPS in intestinal macrophages. CCK‐8 assay showed that LPS induction can significantly decrease the viability of macrophages, whereas CNQX can resist the effects of LPS. The cell viability of the CNQX group was significantly higher than that of the LPS group at the same time‐point (Figure 1A). Apoptosis assay showed that PI and Hoechst 33342 staining showed negative cells in the DMSO group, without significant apoptosis. While the number of positive cells in the LPS group was significantly increased and higher than that in the DMSO group. Besides, the number of positive cells in the CNQX group was down‐regulated and lower than that of the LPS group. Flow cytometry also revealed that CNQX can significantly suppress LPS‐induced macrophage apoptosis, and the apoptotic rate in the CNQX group was significantly lower than that in the LPS group (Figure 1B‐1C). IF staining showed that the expression level of MD2 protein in the DMSO group was relatively low and the fluorescence intensity was weak, whereas MD2 protein expression in the LPS group was significantly up‐regulated, with increased fluorescence intensity, which was significantly higher than that in the DMSO group. Moreover, CNQX can significantly inhibit the protein expression of MD2, and the fluorescence intensity in the CNQX group was weakened and lower than that of the LPS group (Figure 1D). To detect inflammatory factors, we found that LPS can significantly increase the expression of inflammatory factors in intestinal macrophages. To be specific, the levels of IL‐1β, IL‐18 and TNF‐α in the LPS group were significantly increased at 12 h and 24 h, which were higher than those in the DMSO group. While CNQX can inhibit the expression of inflammatory factors. Specifically, the level of inflammatory factors in CNQX group was significantly lower than that in LPS group at 12 h and 24 h (Figure 2A‐2F). The key protein of TLR4 signal showed that LPS can activate TLR4 signal, up‐regulate the expression of TLR4, MyD88 and MD2, which was significantly higher than that of the DMSO group. While CNQX can inhibit the activation of TLR4 signals and down‐regulate the protein expression (Figure 2G‐2H).

### 3.2 The inhibitory effect of CNQX on the mucosal barrier damage of Caco‐2 cells

**FIGURE 3:**
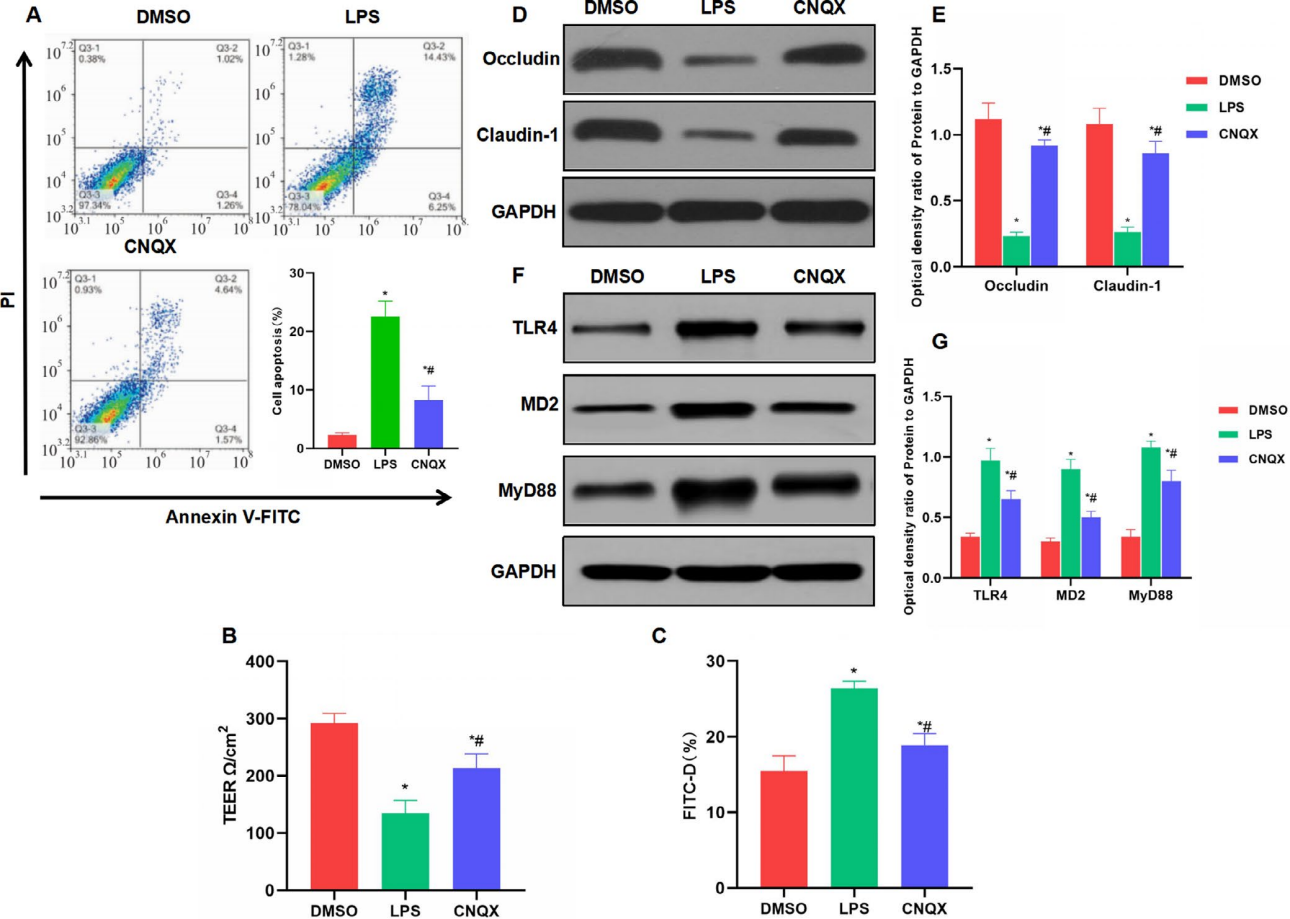
The inhibitory effect of CNQX on the mucosal barrier damage of Caco‐2 cells (A) Detection of apoptosis by flow cytometry ($\bar{x}$ ± s, n = 3): The apoptosis rate of Caco‐2 cells in the LPS group was higher than that in the DMSO group, whereas CNQX can inhibit cell apoptosis. Comparison with the DMSO group, ^*^ *P* < .05; comparison with the LPS group, ^#^ *P* < .05. (B) Results of TEER assay ($\bar{x}$ ± s, n = 3): LPS can down‐regulate the TEER value, which was significantly lower than that of the DMSO group. CNQX can inhibit the effect of LPS and increase the TEER value. Comparison with the DMSO group, ^*^ *P* < .05; comparison with the LPS group, ^#^ *P* < .05. (C) Detection of FITC‐D concentration ($\bar{x}$ ± s, n = 3): The FITC‐D concentration was increased in the LPS group, which was significantly higher than the DMSO group. CNQX can down‐regulate FITC‐D concentration, which was lower than the LPS group. Comparison with the DMSO group, ^*^ *P* < .05; comparison with the LPS group, ^#^ *P* < .05. (D‐E) The expression level of tight junction protein ($\bar{x}$ ± s, n = 3): LPS down‐regulated the expression of tight junction protein (including occludin and claudin‐1). CNQX can inhibit the effect of LPS and increase the expression of occludin as well as claudin‐1. Comparison with the DMSO group, ^*^ *P* < .05; comparison with the LPS group, ^#^ *P* < .05.(F‐G) The expression level of key protein of TLR4 signal ($\bar{x}\;$± s, n = 3): The protein levels of TLR4, MyD88 and MD2 were significantly up‐regulated in the LPS group, which were higher than those of the DMSO group. However, CNQX can significantly inhibit the activation of TLR4 signal and down‐regulate the protein level. For relative expression of protein, comparison with the DMSO group, ^*^ *P* < .05; comparison with the LPS group, ^#^ *P* < .05

In LPS‐induced Caco‐2 inflammation and damage, LPS can significantly induce apoptosis in Caco‐2 cells. Flow cytometry showed that the apoptosis rate of LPS group was significantly higher than that of DMSO group, whereas CNQX can inhibit cell apoptosis, with lower apoptosis rate than that of the LPS group (Figure 3A). TEER assay showed that LPS can down‐regulate the TEER value, indicating that the cell barrier was damaged. While the TEER value in the CNQX group was significantly up‐regulated, which was higher than that in the LPS group (Figure 3B). Similarly, FITC‐D assay also showed that LPS can induce barrier damage and increased the permeability of FITC‐D, whereas the barrier damage was inhibited in the CNQX group (Figure 3C). Western blot assay showed that LPS can down‐regulate the expression of tight junction protein (including occludin and claudin‐1) and activate the TLR4 signal. The expression of TLR4, MD2 and MyD88 was significantly up‐regulated, which was higher than that in the DMSO group. However, the CNQX group can inhibit the activation of TLR4 signal, down‐regulate the expression of TLR4, MD2 and MyD88, increase the expression level of tight junction protein (Figure 3D‐3G).

### 3.3 The effect of CNQX on inflammation and mucosal barrier damage in mice with chronic colitis

**FIGURE 4:**
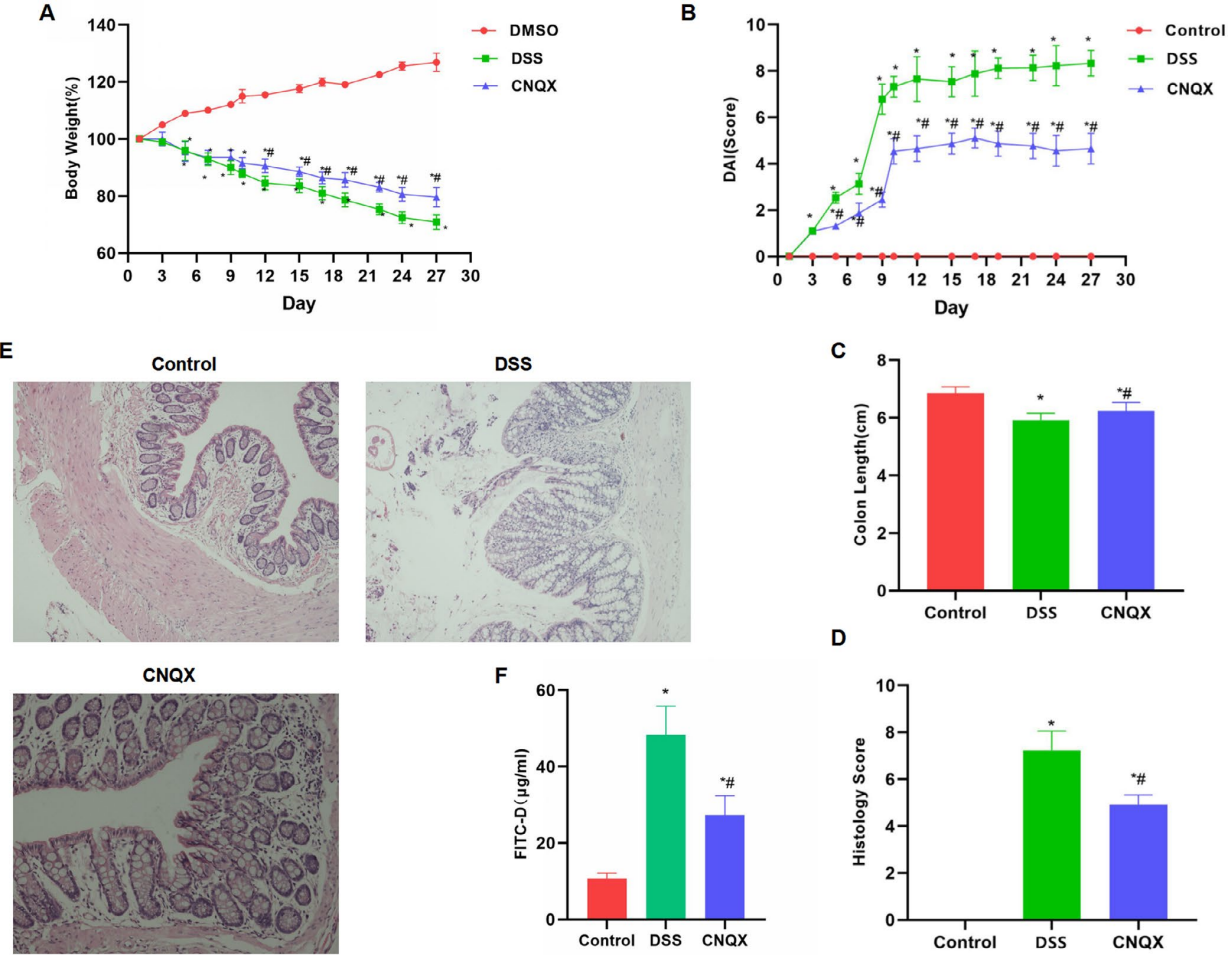
Intervention effect of CNQX on mice with chronic colitis (A‐B) The dynamic observation results of bodyweight and DAI score in mice ($\bar{x}$ ± s, n = 10): The weight of the control group mice increased slowly. However, after DSS intervention, the weight of mice was decreased, which was consistent with the characteristics of colitis. CNQX can improve the living conditions of mice, inhibit bodyweight loss, decrease the DAI score and improve the living standards of mice. Comparison with the Control group, ^*^ *P* < .05; comparison with the DSS group, ^#^ *P* < .05 at the same time‐point. (C‐D) The intestinal length and pathological score of mouse intestinal tissue ($\bar{x}$ ± s, n = 10): Comparison with Control group, ^*^ *P* < .05; comparison with DSS group, ^#^ *P* < .05. (E) H&E staining of mouse intestinal tissue (n = 5): After DSS treatment, the mouse intestinal structure was destroyed, with obvious inflammation and oedema, which was significantly different from the control group. However, the intestinal inflammation was significantly attenuated in the CNQX group. (F) The FITC‐D permeability in mucosal barrier injury ($\bar{x}$ ± s, n = 10): The mucosal permeability of mice was up‐regulated, and the level of FITC‐D was increased in the DSS group. The barrier permeability was decreased, and the level of FITC‐D was down‐regulated in the CNQX group. Comparison with the control group, *P < .05; comparison with the DSS group, #*P* < .05

**FIGURE 5:**
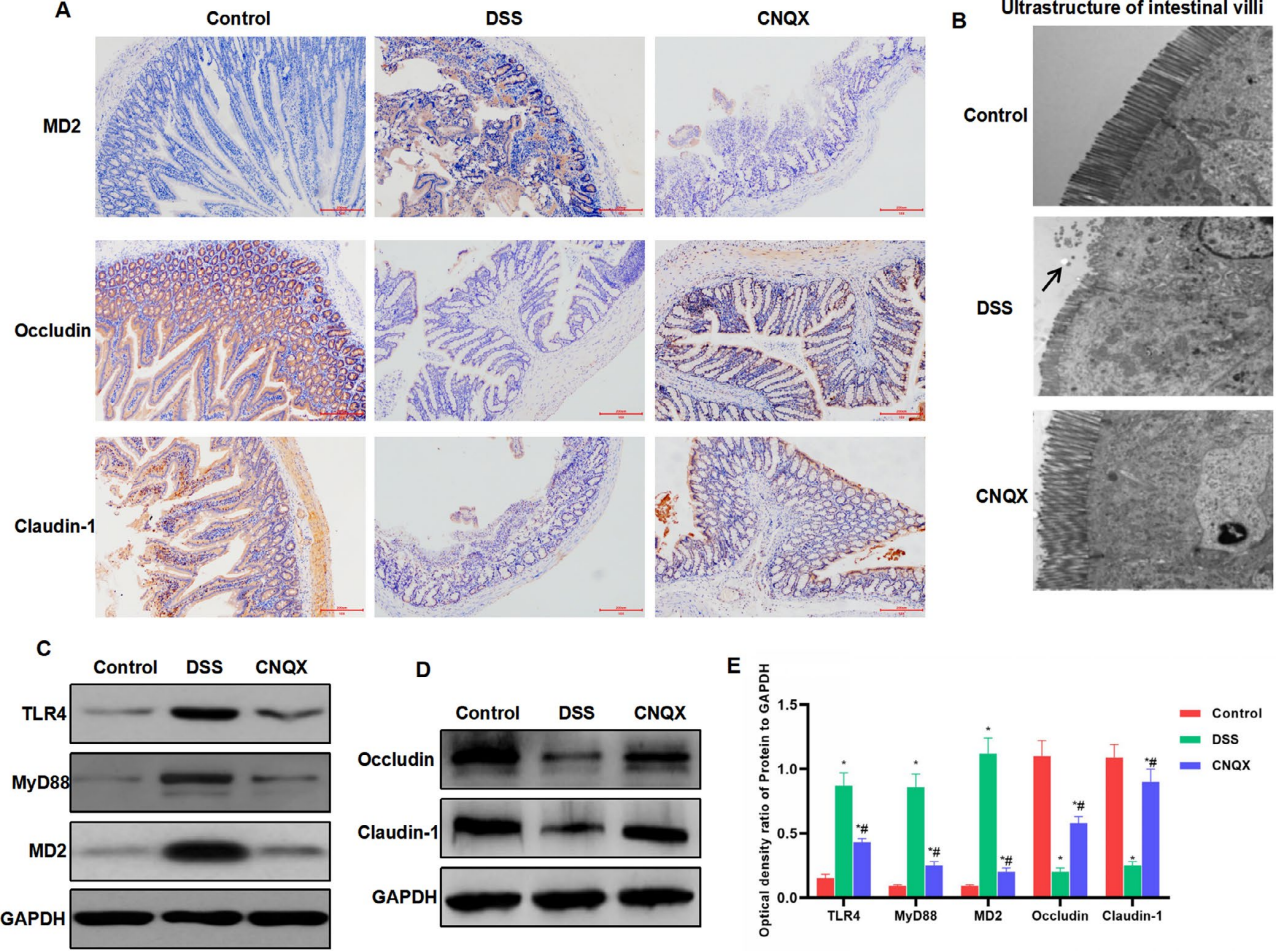
The effect of CNQX on the expression of key protein of the TLR4 signal and tight junction protein (A) The expression of MD2 and tight junction protein by IHC (n = 5): The protein expression of MD2 was relatively low in the Control group, whereas the expression levels of tight junction protein (including occludin and claudin‐1) were higher. The mucosal barrier was destroyed, the expression of MD2 was up‐regulated, and the expression of occludin and claudin‐1 was down‐regulated in the DSS group. CNQX can inhibit the expression of MD2 and up‐regulate the level of occludin and claudin‐1. (B) Results of the ultrastructure of mouse intestinal villi (n = 5): The villi structure was completed and arranged densely in the Control group. The villi structure was damaged, and the length was shortened in the DSS group, which was significantly different from the control group. In the CNQX group, the villi structure was relatively complete, which was significantly different from the DSS group. (C‐D) The effect of CNQX on the expression of key protein of TLR4 signal and tight junction protein ($\bar{x}$ ± s, n = 10): TLR4 signal was activated and the expression of tight junction protein was down‐regulated in the DSS group. CNQX can significantly inhibit the activation of TLR4 signal and improve the expression of tight junction protein. Comparison with the control group, ^*^ *P* < .05; comparison with the DSS group, ^#^ *P* < .05

The bodyweight measurement of mice showed that the weight of mice in the control group was slowly increased. After DSS feeding, the weight of mice was significantly decreased, which was significantly different from the control group. The administration of CNQX can suppress the bodyweight loss, and the bodyweight of mice in the CNQX group was significantly higher than in the DSS group (Figure 4A). The DAI score also showed that the DAI score of the CNQX group was significantly lower than that of the DSS group (Figure 4B). The intestinal length and pathological scores of mice showed that the intestinal inflammation of mice in the DSS group was significant, and CNQX can inhibit the progression of colitis (Figure 4C‐4D). The H&E staining of mouse intestines showed that DSS treatment can destroy the intestinal structure, with obvious inflammation and oedema, which was significantly different from the control group. While the intestinal inflammation of mice in the CNQX group was significantly decreased (Figure 4E). The detection of mucosal barrier damage by FITC‐D revealed that the mucosal barrier of mice in the DSS group was significantly damaged, with up‐regulated concentration of FITC‐D. The concentration of FITC‐D in the CNQX group was significantly lower than that of the DSS group, with inhibited mucosal barrier damage (Figure 4F). IHC assay showed that the MD2 protein level was relatively low in the Control group, whereas the expression of tight junction protein (including occludin and claudin‐1) was relatively high. In the DSS group, the mucosal barrier was damaged, and MD2 expression was up‐regulated, whereas the expression of occludin and claudin‐1 was down‐regulated (Figure 5A). The results of intestinal villi ultrastructure showed that the villi structure in the Control group was completed and arranged densely. The villi structure in the DSS group was damaged, with shortened length, which was significantly different from the control group. In the CNQX group, the villi structure was relatively complete, which was significantly different from the DSS group (Figure 5B). Western blot analysis showed that TLR4 signal was activated in the DSS group, whereas the protein expression of tight junction protein was down‐regulated. CNQX can significantly inhibit the activation of TLR4 signal and increase the level of tight junction protein (Figure 5C‐5E).

### 3.4 Validation of the target binding relationship between CNQX and MD2 protein

**FIGURE 6:**
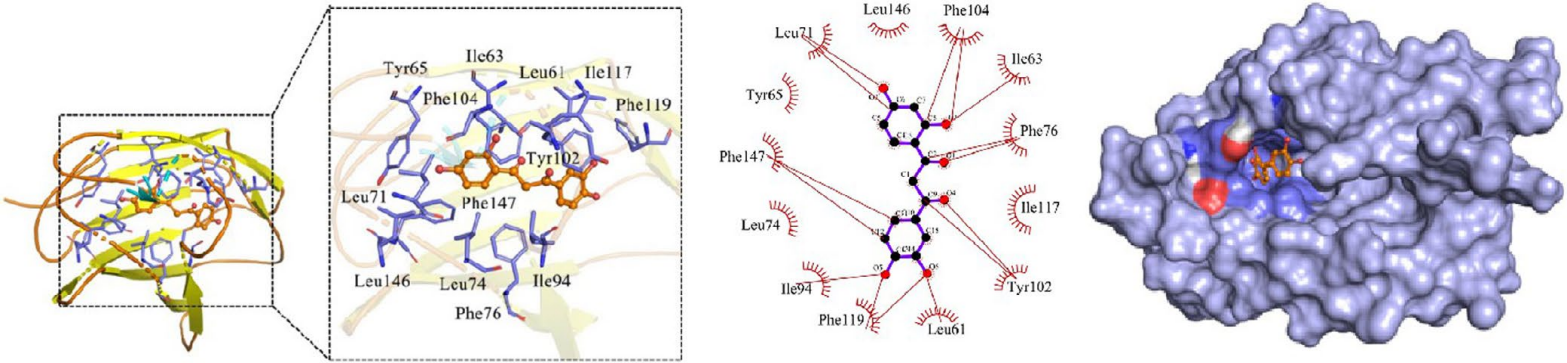
Experimental results of virtual docking between CNQX and MD2 protein

Molecular docking was used to investigate the binding mode between the receptor protein MD2 and the small ligand molecule. The amino acid residues Leu146, Phe104, Ile63, Phe76, Ile117, Tyr102, Leu61, Phe119, Ile94, Leu74, Phe147, Tyr65 and Leu71 can form hydrophobic interactions with small ligand molecules (Figure 6).

**FIGURE 7:**
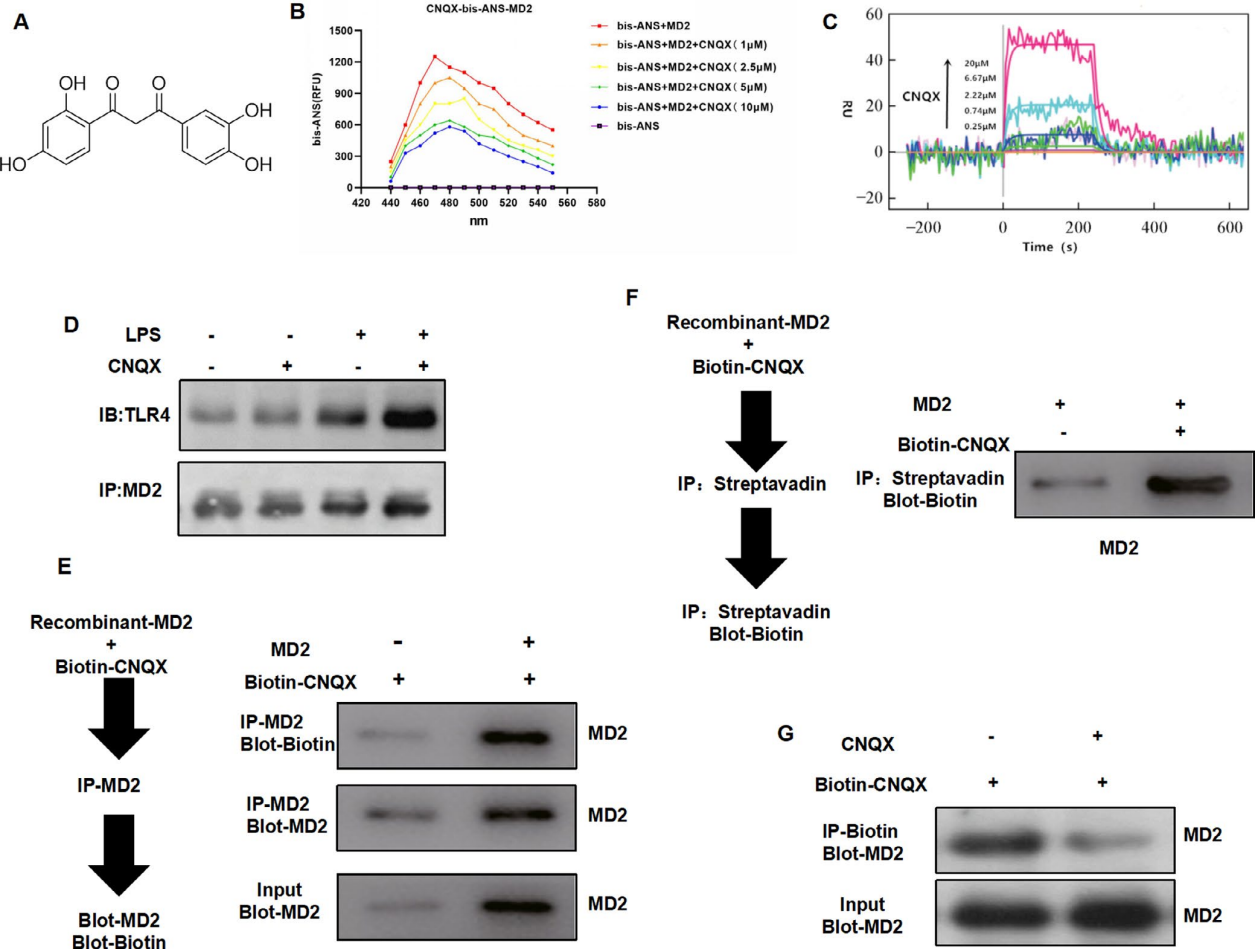
Validation of the target binding relationship between CNQX and MD2 protein (A) The molecular structure of CNQX (B) In the bis‐ANS assay, CNQX can down‐regulate the fluorescence intensity in a dose‐dependent pattern. (C) SPR assay showed that CNQX and MD2 had a targeted binding relationship. (D) Co‐IP assay showed that CNQX had no significant binding relationship with TLR4, but it had a binding relationship with MD2. (E‐F) Pull‐down assay showed that CNQX can bind to MD2. (G) In the competitive binding assay, a large dose of CNQX can compete with Biotin‐Aur and attenuate the binding level of MD2

Bis‐ANS assay showed that Aur can bind to MD2 protein in a dose‐dependent manner (Figure 7A). SPR assay also revealed that MD2 and CNQX had a targeted binding relationship (Figure 7B). Co‐IP assay also showed that Aur can bind to MD2 protein but had no correlation with TLR4 protein (Figure 7C). After Biotin‐Aur labelling, the pull‐down assay showed that MD2 bound directly to Aur, and Aur and Biotin‐Aur had competitive relationship, which also indicated that MD2 was the target protein of Aur (Figure 7D‐7F).

## 4 DISCUSSION

IBD is characterized by repeated attacks, prolonged non‐healing and prone to carcinogenesis. Analysis of clinical data shows that the course of UC is approximately 8‐15 years, with the incidence of tumorigenesis of 22%. Moreover, the incidence of carcinogenesis is 28% for 10‐20 years and is as high as 43% for over 20 years.^15^ Colorectal cancer includes cancerous cases from UC and sporadic cases, which is a common and high‐incidence malignancy, with a 5‐year survival rate of less than 50%.^16^ Therefore, the prevention of IBD is particularly important. The conformational study of TLR‐4 and LPS revealed that the TLR‐4 agonist LPS can indeed promote the oligomerization of MD‐2‐TLR‐4 and increase the expression level of TLR‐4 in the cell membrane^17^, ^18^. While the TLR‐4 antagonist inhibits the oligomerization of MD‐2‐TLR‐4 and decreases the expression of TLR‐4 in the cell membrane, thereby attenuating the activation of NF‐κB and inhibiting the occurrence of inflammatory responses.^19^, ^20^ Although TLR‐4 can recognize and bind with LPS, the stability and expression level of TLR‐4 distribution in the cell membrane, as well as the activation of downstream pathways, depend on the formation of a complex with its co‐receptor MD‐2. MD‐2, glycoprotein with 20‐25 kD in size, forms a complex with TLR‐4, that is TLR‐4‐MD‐2, which is located on the cell membrane. When LPS binds to TLR‐4‐MD‐2, the two molecules, LPS‐TLR‐4‐MD‐2, form a dimer (oligomerization) and undergo conformational rotation, which is stably expressed in the cell membrane.^21^ Knockout of MD‐2, inhibition of the formation of MD‐2‐TLR‐4 complex or suppression of the complex oligomerization can all attenuate the expression of TLR‐4 in the cell membrane.^22^ Therefore, LPS‐induced oligomerization of TLR‐4‐MD‐2 complex is an essential condition for the high expression of TLR‐4 in the cell membrane, revealing that MD2 is a key protein for TLR4 signalling and NF‐κB activation.^23^

Our team has previously revealed the up‐regulated expression of MD2 in various IBD, including UC and CD, and the high expression of MD2 is closely associated with the massive expression of inflammatory factors.^12^ Suppression of MD2 level can inhibit the activation of TLR4 signalling and inhibit mucosal barrier damage caused by inflammation. Additionally, our previous study has also demonstrated that aureusidin is an effective inhibitor of MD2 to suppress inflammation.^14^ In this study, we modified the structure of aureusidin and found that CNQX had anti‐inflammatory effects. Intestinal macrophages are important regulatory cells for the intestinal immune microenvironment. LPS can induce the activation of intestinal macrophages and the expression of TLR4 signals. A large number of inflammatory factors (including TNF‐α, IL‐18 and IL‐1β) can induce intestinal mucosal damage and up‐regulation of permeability.^24^ The CNQX intervention can down‐regulate the inflammatory response of intestinal macrophages. More importantly, CNQX can down‐regulate the level of MD2 protein and activate the expression of inflammatory factors. Caco‐2 cells were further used for the mucosal barrier in vitro. As a result, we found that LPS can induce Caco‐2 damage. FITC‐D and TEER assays also showed that LPS destroyed the mucosal barrier and improved permeability, which is associated with the activation of TLR4. CNQX pre‐treatment can significantly inhibit the inflammatory damage of Caco‐2 cells, decrease the permeability of the mucosal barrier, increase the TEER value and decrease the concentration of FITC‐D. Therefore, through intestinal macrophages and Caco‐2 cell models, we found that CNQX can inhibit MD2‐mediated TLR4 signal activation to exert anti‐inflammatory effects. In the study of the mechanism, we also validated that CNQX had a targeted binding relationship with MD2, which were further proved by Bis‐ANS, SPR and other assays. To explore the role of CNQX in IBD in mice, IBD mice were established by DSS treatment. DSS can destroy the intestinal mucosa of mice and obviously damage the mucosal barrier of mice, which is manifested as the down‐regulated expression of tight junction protein (occludin and claudin‐1). Meanwhile, TEM can reveal the severe damage of the structure of mouse intestinal villi.^25^, ^26^ DSS can induce the activation of TLR4 signal in the mouse intestine. CNQX can significantly alleviate this damage and attenuate he inflammatory response in the mouse intestine. FITC‐D test showed that CNQX can decrease the permeability of the mucosal barrier. The electron microscope observation of the villus structure also showed that CNQX can inhibit mucosal damage. Most importantly, CNQX can increase the expression of tight junction protein (occludin and claudin‐1), which is also one of the important markers of the inhibited mucosal barrier damage.

## 5 CONCLUSION

In this study, we found that the aureusidin derivative CNQX is an effective inhibitor of MD2, which can inhibit the activation of TLR4 and downstream signals by targeting MD2, thereby exerting anti‐inflammatory effects. In the study of IBD, CNQX can also inhibit mucosal barrier damage and inflammation. CNQX is a novel type of MD2 protein inhibitor, which is worthy of further research and development.

## CONFLICT OF INTEREST

No Competing interests.

## AUTHOR CONTRIBUTION

**yi yang:** Conceptualization (equal); Investigation (equal); Methodology (equal). **yongjia sheng:** Investigation (equal); Resources (equal); Supervision (equal). **jin wang:** Formal analysis (equal); Resources (equal); Validation (equal); Visualization (equal). **xiaohong zhou:** Conceptualization (equal); Funding acquisition (equal); Investigation (equal). **qiaobing guan:** Visualization (equal); Writing‐original draft (equal). **heping shen:** Methodology (equal); Supervision (equal); Writing‐review & editing (equal). **wenyan Li:** Conceptualization (equal); Project administration (equal); Resources (equal); Visualization (equal). **shuiliang ruan:** Writing‐original draft (equal); Writing‐review & editing (equal).

## ETHICAL STATEMENT

Ethical Approval and Consent to participate: The study approved with Ethics Committee.

## CONSENT FOR PUBLICATION

All authors approval published the article.

## Data Availability

The data that support the findings of this study are available from the corresponding author upon reasonable request.
