# Correction to Aureusidin derivative CNQX inhibits chronic colitis inflammation and mucosal barrier damage by targeting myeloid differentiation 2 protein

**DOI:** 10.1111/jcmm.18117

**Published:** 2024-03-17

In Yi Yang et al^1^, in the published version of the article, Figure 5A encountered an error during typesetting. The correct figure is shown below. The authors confirm all results, and conclusions of this article remain unchanged.

**FIGURE 5:**
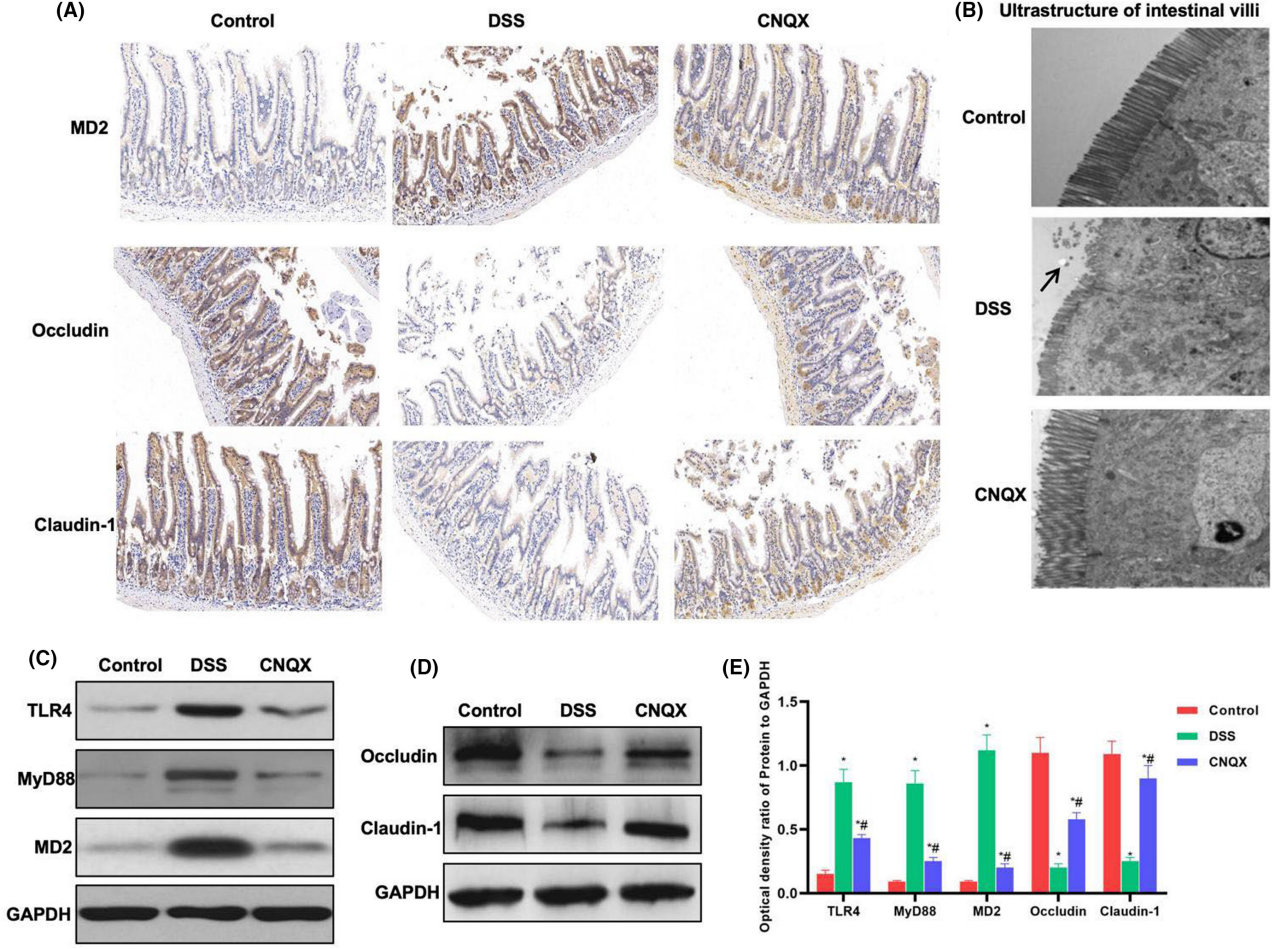
The effect of CNQX on the expression of key protein of the TLR4 signal and tight junction protein (A) The expression of MD2 and tight junction protein by IHC (*n* = 5): The protein expression of MD2 was relatively low in the control group, whereas the expression levels of tight junction protein (including occludin and claudin‐1) were higher. The mucosal barrier was destroyed, the expression of MD2 was up‐regulated, and the expression of occludin and claudin‐1 was down‐regulated in the DSS group. CNQX can inhibit the expression of MD2 and up‐regulate the level of occludin and claudin‐1. (B) Results of the ultrastructure of mouse intestinal villi (*n* = 5): The villi structure was completed and arranged densely in the control group. The villi structure was damaged, and the length was shortened in the DSS group, which was significantly different from the control group. In the CNQX group, the villi structure was relatively complete, which was significantly different from the DSS group. (C–E) The effect of CNQX on the expression of key protein of TLR4 signal and tight junction protein (x ± s, *n* = 10): TLR4 signal was activated, and the expression of tight junction protein was down‐regulated in the DSS group. CNQX can significantly inhibit the activation of TLR4 signal and improve the expression of tight junction protein. Comparison with the control group, *p < 0.05; comparison with the DSS group, #*p* < 0.05.
